# Alcohol policy, multi-level governance and corporate political strategy: The campaign for Scotland’s minimum unit pricing in Edinburgh, London and Brussels

**DOI:** 10.1177/1369148120959040

**Published:** 2020-11-03

**Authors:** Benjamin Hawkins, Jim McCambridge

**Affiliations:** 1 London School of Hygiene & Tropical Medicine, London, UK; 2 Department of Health Sciences, University of York, York, UK

**Keywords:** alcohol industry, alcohol policy, health policy, multi-level governance, MUP, Scotland

## Abstract

The Scottish government’s plans for a minimum unit price for alcohol were vehemently opposed by the alcohol industry leading to a 6-year delay in implementation after legislation was passed. This article seeks to explain the consequences of devolution and European Union membership for the development of minimum unit price in Scotland through the concepts of multi-level governance, veto points and venue shifting. Systems of multi-level governance create policy interdependencies between settings, an increased number of veto points at which policies can be blocked, and the potential for policy actors to shift decision-making to forums where favourable outcomes are more likely to be attained. In the minimum unit price debates, the alcohol industry engaged in multiple forms of venue shifting and used regulatory compliance procedures and legal challenges at the EU level to try to prevent and delay the policy. This has led to a ‘chilling effect’ on subsequent alcohol policy developments across the United Kingdom.

## Introduction

Scotland experiences significant alcohol-related health inequalities internally and in comparison with the rest of the United Kingdom and Europe (Fox, 2013; Leon and McCambridge, 2006; McCartney et al., 2011). Reducing alcohol-related harms was identified as a key objective of the Scottish National Party (SNP, 2007) government elected in 2007. The international research consensus indicates that the most effective and cost-effective ways of addressing alcohol-related harms are via population level measures that restrict alcohol’s availability and marketing and increase price (Babor et al., 2010; see also World Health Organization (WHO), 2017). However, these measures are vehemently opposed by vocal and economically powerful sections of the global alcohol industry (Hawkins and Holden, 2013; McCambridge et al., 2014a).

The Scottish government’s 2008 alcohol strategy sought to implement evidence based policies including a minimum unit price (MUP) for alcohol (Scottish Government, 2008). This represented a seminal moment in UK alcohol policy and a point of divergence from the prevailing Scottish and UK governments’ approach, which had focussed – and in England continues to focus – on less effective, but industry-favoured, policy alternatives based on self-regulation, targeted interventions and consumer responsibility within an overall framework of partnership between government and industry (Anderson, 2007; Hawkins et al., 2012).

Having failed to pass MUP into law as a minority government via the Alcohol Etc. (Scotland) Act in 2010, the majority SNP administration, elected in 2011, reintroduced MUP legislation to the Scottish Parliament and the Alcohol (Minimum Pricing) (Scotland) Act received Royal Assent in June 2012. This was followed by the Alcohol (Minimum Price per Unit) (Scotland) Order 2013, which set the level of MUP at 50p per unit. In March 2012, MUP was adopted in the UK government’s alcohol strategy for England (HM Government, 2012) to the great surprise of many policy makers and advocates (Hawkins and McCambridge, 2020a). However, it was announced in July 2013 that the government would not bring forward measures to implement the policy, citing the need for more evidence of its effects, a decision that was strongly criticised by public health actors (Godlee, 2014). While there have been no further developments on MUP in England, the Welsh government (2018) announced in October 2017 that it too would enact MUP. Similar proposals in Northern Ireland have been delayed by the ongoing suspension of the power-sharing executive in Belfast since January 2017. This policy ‘spillover’ to other parts of the United Kingdom, albeit abortive in England, underlines the wider implications of Scottish alcohol policy debates, and thus explains in part the strength of industry opposition to the implementation of MUP there.

The politics of MUP in Scotland must be understood in the context of the wider contextual and constitutional factors in which the policy developed – that is, the United Kingdom’s asymmetrical system of quasi-federalism and its membership of the European Union (EU) – which structure the policy process (Holden and Hawkins, 2012; Katikireddi and McLean, 2012; Smith and Collin, 2013). The devolution settlement meant that tax-based measures (set UK-wide at Westminster) were unavailable to the Scottish government, but MUP could be introduced as a (devolved) health measure (Holden and Hawkins, 2012). MUP thus exemplified the ability of policy makers in Edinburgh to develop specifically Scottish policy initiatives within the confines of the restricted competences devolved to Edinburgh in 1999 (Holden and Hawkins, 2012). MUP was thus connected to the SNP’s agenda for Scottish independence from the United Kingdom. At the same time, policy divergence between Scotland and England emerged as a potential source of tension between Scottish and UK governments and between the UK-level and Scottish branches of the main political parties.

As well as the internal politics of the post-devolution United Kingdom, policy processes at the EU level played an important role in the MUP process (see Katikireddi and McLean, 2012). Under the EU’s technical standards directive, member-states must notify the Commission of the EU (from here on ‘the Commission’) of their intention of adopting policy measures which have potential implications for the functioning of EU law, so that the Commission and other member-states may raise any issues or objections. Furthermore, the potential exists for formal legal challenges to be brought against measures believed to be non-compliant with EU law.

Once industry lobbying failed to prevent the passage of MUP into law, and proposals for the implementation of a 50p per unit minimum price were brought forward by the Scottish government in June 2012, the Scotch Whisky Association (SWA) launched a case for judicial review of the policy in July 2012, arguing that MUP breached EU single market and competition law. The Outer House of the Court of Session in Edinburgh found in favour of the Scottish government in May 2013; a decision which was appealed by the SWA to the Inner House (the Scottish Court of Appeal). The case was then referred by the Inner House in April 2014 to the Court of Justice of the European Union (CJEU) for a preliminary ruling on the relevant points of EU law. The CJEU opinion, issued in December 2015, stated that it was for the national courts to be the ultimate arbiters of the case. In light of this, the Inner House confirmed the judgement of the Outer House that MUP was legal in October 2016. In December 2016, the SWA was granted leave to appeal the decision of the Scottish courts to the UK Supreme Court. In its ruling in November 2017, the court again found in favour of the Scottish government, finally exhausting the legal avenues open to the SWA in opposing the policy. MUP came into effect in May 2018; a delay of 6 years between legislation and implementation.

This article examines the consequences of devolution and EU membership for the development and implementation of alcohol pricing policy in Scotland, and the strategies of alcohol industry actors to oppose these developments. We examine Scottish policy making as part of a complex system of multi-level governance including the United Kingdom and EU levels. The case of MUP offers an interesting case study of the dynamics of multi-level governance and the potential of multiple decision-making forums to both facilitate and stymie policy development. On the one hand, devolution, and the creation of new polities such as that centred around the Scottish Parliament and Scottish government, create additional sites of policy entrepreneurship which may spread horizontally and vertically to other decision-making forums (Holden and Hawkins, 2012). On the other hand, the interconnectedness between different levels of governance may create additional veto points at which such policy entrepreneurship, particularly at lower levels of the constitutional structure, may be opposed and potentially halted (Hawkins and Holden, 2016).

The constitutional-structural factors identified above placed potential limitations on the ability of the Scottish government to act given the need for compliance with both the distribution of powers between Edinburgh and London and with EU single market and competition laws. This created the need for policy advocates to lobby in favour of the policy and gain political buy-in from actors in Edinburgh, Westminster and Brussels. The court case on MUP in Luxembourg, in particular, created inter-dependencies between the Scottish and UK governments, not least because the United Kingdom (as the EU member-state) had formal standing at the CJEU rather than the Scottish government. The complexities of the MUP process demonstrates the capacity of trans-national corporations (TNCs) to operate in co-ordinated ways across levels of governance and jurisdictions. It underlines also the strategic challenges facing multi-level and trans-national non-governmental organization (NGO) networks in supporting government and countering industry opposition to achieve important policy changes.

## Methods

The analysis presented here draws on the key concepts of ‘multi-level governance’ and the related ideas of ‘veto points’ and ‘venue shifting’ which have been employed elsewhere in order to understand corporate influence over health policy (Hawkins and Holden, 2016; Holden and Hawkins, 2016) including in the context of EU single market law (Holden and Hawkins, 2018). For this reason, we do not undertake an exhaustive review of this literature here. In addition, the current article is an example of ‘problem driven’ research in that the primary aim is to elucidate and understand a specific empirical case and the application of the theories identified above is designed to facilitate this enquiry (Howarth, 2000). The literature discussed below was identified purposively on the basis of its relevance for the current research questions, namely how multi-level governance structures within the context of EU member-states with subnational federal or devolved administrations. In particular, we situate the current article in relation to those studies focussing on the United Kingdom and/ or health policy in this context. The logic of the study from which this article is drawn is inductive; with explanations sought for the key themes emerging from the data within relevant bodies of theoretical literature. The contribution of the article is, therefore, primarily empirical rather than theoretical. However, the study offers important insights into multi-level governance and contributes to the associated literature in ways which will be of relevance and interest to policy scholars beyond the alcohol field.

The concept of multi-level governance has been discussed at length in the context of European integration to identify the ways in which policy making occurs in multiple forums, and via networks of policy actors at the subnational, national and supra-nations levels of governance, recognising that policy developments in one context have important implications for those elsewhere (Hooghe and Marks, 2001, 2003; Marks et al., 1996; Marks and Hooghe, 2004).

A related literature has applied the concept of multi-level governance to the United Kingdom (Bache and Flinders, 2004) particularly in the context of debates about the ‘Europeanisation’ of UK politics and policy making (Bache, 2008; Bache and Jordan, 2006; Radaelli, 2008). However, the application of the concept of multi-level governance to the United Kingdom was a response not just to EU membership, but to the consequences of devolution for UK politics. This has led to recognition of a distinctively Scottish policy ‘style’ (Keating et al., 2009; see also Holden and Hawkins, 2012), which, it is argued, has in turn impacted policy making at the UK level (Cairney, 2008, 2011).

Of particular relevance to the present article are the small number of studies which have engaged with the concept of multi-level governance in the context of public health policy in the United Kingdom (Katikireddi et al., 2016), most notably in the fields of tobacco control (Asare et al., 2009; Cairney, 2007; Cairney et al., 2011). These studies trace the evolution of tobacco control policy in the United Kingdom via developments at the EU, United Kingdom and devolved (Scottish) levels, identifying overlapping, ambiguously divided competences in tobacco policy between levels of governance. Their focus is largely on the ways in which policy can be advanced, and the opportunities for policy innovations within each context, while acknowledging the constraints which activity at one level (mainly the EU) may have for that elsewhere.

A more limited literature has focussed on the implications of EU law for alcohol pricing policy (Holden and Hawkins, 2018; Katikireddi and McLean, 2012). According to these studies, the EU both creates opportunities for advancement on alcohol (and wider public health) policy through the regulatory capacity of the European institutions, and poses potential threats to domestic policy agendas which must comply with, and can be challenged under, EU law (Holden and Hawkins, 2018).

Recent studies have identified how tobacco industry actors employ highly integrated global strategies to shape policy environments and engage in processes of venue shifting within multi-level governance systems (Hawkins et al., 2018, 2019). Given the similarities identified elsewhere between the market and political strategies of the global tobacco and alcohol industries, it is likely that alcohol industry actors adopt similar approaches in this regard (Hawkins et al., 2016; McCambridge et al., 2019a, 2019b).

The current article adds to these studies by examining the capacity of policy actors such as alcohol corporations to use multiple decision-making venues within multi-level systems of governance to stymie policies that run counter to their interests. Following previous studies of the global tobacco industry (Hawkins and Holden, 2016), it draws on veto player theory (Tsebelis, 2002) to understand the ways in which the post-1999 UK constitutional settlement – encompassing devolution and EU membership – created additional hurdles which policies such as MUP must overcome in order to be adopted. More specifically, we employ the concept of ‘veto points’ to denote key decision making junctures in the policy process through which policy proposals such as MUP must successfully pass in order to be enacted, but at which their passage can be halted by the decisions of a relevant actor (Hawkins and Holden, 2016; Immergut, 1992). This includes, for example, the ruling of a constitutional or other court about the compatibility of a law with the constitution or pre-existing national laws. Complex systems of multi-level governance, particularly highly-developed legal orders such as the EU in which the compatibility of laws at different levels of governance must be ensured, offer a multiplicity of potential veto points (Holden and Hawkins, 2016).

The article also draws on the concept of ‘venue shifting’ to explain attempts by policy actors to move decision making to settings in which they are more likely to achieve their policy objectives (Baumgartner and Jones, 1991, 1993; Coen, 2007; Mazey and Richardson, 2006). In this article, we use the concept to refer not just to attempts to shift the venue of decision making between different levels of governance (e.g. from the national to the supra-national (EU) level), but also to the attempt to shift authority from the political to the legal arena through judicial review of MUP. In addition, we identify how industry actors sought to involve different ministries in government and Directorates General within the Commission in a form of intra-institutional venue shifting.

While previous studies have noted the opportunities afforded to the global alcohol industry by EU law to block unfavoured policies and engage in venue shifting in the context of MUP (Holden and Hawkins, 2018), the current article moves beyond these to analyse the micro-politics of the MUP legislative and implementation processes. It analyses the ways in which policy process at the EU level, including legal challenges to MUP, affected the activities and decision-making of the Scottish government. In so doing, it highlights the integrated nature of different aspects of industry strategy and the significant opportunity costs this imposes on governments, which must respond to multi-faceted industry strategies across multiple venues.

The article draws on 26 semi-structured interviews (Brinkmann, 2013; Rubin and Rubin, 2012), conducted by the first author with policy actors in London and Edinburgh between February and October 2018. Respondents included civil servants and government actors (n = 8) from relevant ministries and agencies, members of the UK Parliament (n = 1) and the Scottish Parliament (n = 1), civil society actors (from alcohol-related NGOs, medical associations and public health bodies; n = 13) and academic researchers (n = 3). These were identified via purposive sampling (based on stakeholder mapping) (Brugha and Varvasovszky, 2000; Varvasovszky and Brugha, 2000) and snowball sampling.

In a departure from previous studies of the roles of the alcohol industry in UK alcohol policy, the decision was taken at the outset not to undertake interviews with alcohol industry representatives for a number of reasons. These included uncertainties about both gaining access and the additional complexity anticipated in the data in light of previous findings, which have exposed industry strategy in ways which industry actors would likely perceive to be detrimental to their interests (Hawkins and McCambridge, 2014; Lyness and McCambridge, 2014; McCambridge et al., 2014b). The accumulation of research attention paid specifically to the industry has led industry actors to politicise academic alcohol policy research (House of Lords, 2015). This context means data generated from interviews with industry actors would now have a different status from that obtained from them in previous studies (and from other policy actors in the present study) because the interviews are more likely to be used by industry respondents as opportunities to frame perceptions about policy debates and their activities in ways in keeping with their information management and wider political strategies (McCambridge et al., 2018). An important implication of this decision is that the dataset is restricted to other actors’ perceptions of industry strategy in relation to the wider policy processes being studied. Methodologically, this data limitation precludes a focus on internal industry dynamics and requires triangulation of perspectives from interviewees from different sectors, including those who had engaged with and worked closely with industry actors.

Interviews were recorded, transcribed and analysed using established qualitative research methods. A two-stage process of thematic coding (Braun and Clarke, 2006) was undertaken by the first author in liaison with the second author who reviewed transcripts independently to identify key topics and themes. The analysis was subsequently refined in an iterative process led by the first author. This emerges from a wider study of UK alcohol policy across the time period examined here. As such, the data collection (i.e. interviews) and data analysis (i.e. thematic coding) processes were undertaken across a range of issues. In keeping with the principally empirical aims of this study, the presentation and analysis of interview material is organised in terms of the key themes and issues emerging around the implementation of MUP in Scotland, rather than around aspects of the theories we employ to elucidate and explain this case. We return to, and explain the relevance of, the data presented, in our discussion of the theoretical contribution made by this study.

The terms ‘state’ and ‘nation’ are often used in different ways in different contexts within political science and international relations, including in the context of European integration studies. This is particularly the case when discussing federal systems. In the United States (US), for instance, the federal level is often referred to as ‘the nation’ while the constituent parts of the federation, as the name of the country suggests, are ‘states’. In Spain, meanwhile, the state is made up of various ‘nationalities’ enjoying varying degrees of autonomy. Defining ‘the nation’, and what counts as the national level, is thus a highly political act, which speak to vital issues of political identity. To avoid this controversy, we employ the terms ‘sub-state’ and ‘state’ levels policy making to refer to policy decisions taken in Scotland and the United Kingdom, respectively. In keeping with the terminology current in European integration studies, we do, however, employ the term ‘supra-national’ to refer to political activity at the EU level.

## Industry lobbying at the Scottish and UK levels

We identified and differentiated industry influencing strategies, in terms of the specific methods employed, which reflected the distribution of policy competences and political dynamics at each level of governance. At the Scottish and UK levels, industry actors employed policy influencing strategies in line with those identified by previous studies. These have highlighted the importance of re-framing the nature of alcohol-related harms as a public health issue to legitimise whole population measures, and MUP in particular, as policy instruments in Scotland (Hawkins and Holden, 2013; Katikireddi et al., 2014). While alcohol harms generate a large health burden, the hegemonic hold of industry perspectives at the UK government level meant such a re-framing was necessary, and had important political consequences. With the support of the then Cabinet Secretary for Health and Wellbeing and Deputy First Minister, Nicola Sturgeon, the policy had key political buy-in at the highest level of government (Hawkins and McCambridge, 2020a, 2020b). Policies relevant to addressing alcohol harms extend, however, beyond the remit of health ministries, creating the potential for tensions to occur with other parts of the government engaged in alcohol policy. These offer opportunities which could be exploited by industry actors seeking to undermine the policy. The use of cross-departmental lobbying within the Scottish government by the alcohol industry during the passage of the 2010 legislation has been detailed previously, along with attempts to shift the decision making ‘venue’ to Westminster by redefining alcohol pricing policy as a tax issue (Holden and Hawkins, 2012). We refer to attempts to play off the competing priorities of decision makers in different policy areas (e.g. between health and trade ministries) against one another as ‘intra-institutional’ venue shifting, and attempts to shift decision making to different levels of policy making as ‘multi-level’ venue shifting.

After 2011, the approach to both ‘intra-institutional’ and ‘multi-level’ venue shifting evolved as industry strategies adapted to developments in the policy process. Industry actors sought to exploit tensions both between the Scottish and Westminster governments, and between government departments in London with different alcohol policy responsibilities, given the potential non-alignment of priorities across ministries and between levels of government. For example, the UK Department for Business Innovation and Skills (BIS),^1^ concerned with supporting the international trade interests of UK businesses, including the alcohol industry, will see alcohol policy in very different terms to the Health and Social Care Directorates of the Scottish government tasked with improving health outcomes. However, despite these efforts Scottish government respondents agreed that the industry enjoyed limited success in influencing the policy through these channels.

Tensions between governance levels had been evident in the reluctance of the Labour Party to support the introduction of MUP at Westminster. This was, at least in part, a result of pressure from the Scottish Labour Party not to be seen to endorse the SNP’s flagship policy in Scotland and to instead support the existing position of the party towards MUP in Edinburgh. Industry actors also sought to identify potential splits within the SNP, and between the Scottish and UK level parties. As a former MSP commented, the industry hoped SNP MPs at Westminster, may be concerned about the economic interests of Scotland as a whisky producer, and a potentially negative image of an iconic Scottish brand being projected globally by MUP:

You know, [the whisky industry] always had that [hold over] politicians in Scotland [. . .] my colleagues in Westminster still . . . ‘Scotch Whisky Association, support brand Scotland’, you know. They’ve worked it. And they [the industry] just think Scottish politicians are there to [do] what they say.

In keeping with previously identified strategies (Hawkins and Holden, 2014), the industry targeted influential policy actors with whisky interests in their constituencies – including the Leader of the SNP at Westminster, Angus Robertson.

Industry strategies at the Scottish and UK levels are indicative of the ways in which industry actors engage in processes of venue shifting within systems of multi-level governance in ways which are cognizant of the specific constitutional and political dynamics at each level. These strategies are designed to move decision making to the locus in which a favourable policy outcome can most likely be achieved. Despite industry attempts to play off potentially competing interests between governance levels, policy remits and the competing priorities of individual policy actors, the Scottish and UK governments demonstrated ongoing political commitment to MUP in Scotland throughout the legislative process and subsequent legal challenge.

## Policy influencing at the EU-level

Perhaps the most obvious example of venue shifting was the attempt by industry actors to move decisions on the legality of MUP to the EU level by testing its compatibility with EU law in court. Within the EU, public policies within one member-state have potential implications for both other members and the functioning of the common EU policies, most notably those pertaining to the internal market. Consequently, the EU operates a system in which, subject to certain criteria, member-states must notify the Commission (and thereby other member states) about proposed changes to standards and regulations, with the objective of preventing the introduction of technical barriers to trade.

This procedure is set out initially in the Technical Standards and Regulations Directive (TSRD) 98/34/EC (in force at the time of the MUP process and subsequently amended by Directive 2015/1535/EU). It provides for a 3-month scrutiny period and the submission of detailed opinions of key actors within the EU (i.e. the Commission and other member-states). While this process cannot formally veto domestic legislation, there is a norm of consensus-based decision making within the EU, and an awareness among governments that they also may need to seek subsequent changes to domestic laws in other member-states in areas of national interest. Furthermore, the potential for more formal legal challenges to policies once enacted means that this consultation process has significant political force and governments seek to accommodate concerns raised wherever possible in an attempt to avoid subsequent litigation (see Holden and Hawkins, 2018).

There was some uncertainty within the Scottish government about whether the proposed Alcohol (Minimum Price per Unit) (Scotland) Order 2013 needed to be referred to the Commission under the TSRD. However, given the industry’s clearly articulated intention to launch legal challenges to MUP, the decision was taken to refer the Order to the Commission on 25 June 2012 (Ref: 2012/0394/ UK). As a representative of the Scottish government explained:

We thought it was actually debatable as to whether it met the qualifying criteria under the technical standards directive but we thought that some parts of the Commission would fight us on that point of principle [. . .]. And we believed that we had the evidence and the argument to be able to win at the end of the day. [. . .] We clearly wanted the right result. We wanted to win, and we didn’t want to do anything quick if that jeopardised eventual success. But we were not playing for time and we were trying to do it as quickly and efficiently as possible so we took that on the chin and entered into the technical standards process.

The TSRD notification procedure represented an opportunity for the Scottish government to identify and resolve any potential legal issues and achieve political compromise before implementation, thereby increasing their chances of success in any subsequent legal challenges. Failure to refer could be used by the industry as grounds for further, time-consuming procedural challenges. The decisions taken by the Scottish government here demonstrate the indirect effects of the ongoing threat of legal challenges throughout the legislative process (Hawkins and McCambridge, 2020b). The prospect of legal challenges appears to have shaped government thinking on referring the measure to the Commission, thereby delaying the adoption of legislation and creating additional regulatory hurdles at which the policy could potentially be halted.

The example of the TSRD process highlights also the way in which specific constitutional features within a system of multi-level governance can both facilitate and stymie policy development. On the one hand, the referral process is designed to facilitate the emergence of locally specific policy regimes in keeping with the EU’s principle of subsidiarity, through the identification and resolutions of potential barriers to implementation at an early stage in the policy process. Yet the need primacy of EU law, and the existence of a formalised compliance mechanism, creates a potential veto point at which policies emanating from lower levels may be challenged. As is evident in the following section, this creates the potential for well-resourced policy actors, such as those in the alcohol industry, to shape policy debates at a crucial stage of the process. The ability to feed into the early formation of policy proposals creates path dependencies and is thus a powerful means of shaping subsequent development.

## Industry lobbying in the TSRD process

The TSRD notification procedure represented a potentially significant barrier for MUP to overcome, given the number of member-states which are alcohol producers, the relative power of the ‘pro-business’ Directorates General (i.e. DG Trade; DG Internal Market) within the Commission and the extensive alcohol industry connections both there and with governments of alcohol-producing states. As one Scottish civil servant with knowledge of the process commented:

[The industry spent] a lot of time spent over in Brussels talking to the DG [Competition], and other parts of the Commission, to try and get them to say what we were doing was contrary to European law. [. . .] They were doing the rounds in Brussels, and it was very much about, well, the Scottish Government are doing something that’s contrary to competition law. So, they were trying to get the UK Government to stop what we were doing, they were trying to get the European Commission to stop what we were doing.

Recognition of Industry expertise in navigating the policy terrain in Brussels, was echoed by another Scottish government civil servant who underlined the extent of connections and expertise in EU level policy work within the alcohol industry:

we were aware that they were active in Brussels lobbying and indeed they have real experience in doing so. In many ways the creation of the Scottish Whisky Association was [. . .] to allow the industry to address trade barriers so they have literally decades of experience of this, so we knew that they were working Brussels and would be doing so in an expert fashion.

In addition to targeting the Commission, respondents identified that industry trade associations sought to highlight potential issues with MUP to the governments of alcohol producing member-states in the hope they would raise objections to the law during the notification process. This strategy appeared to yield some success. Respondents cited the case of Poland, which was reported to have reproduced statements by the Polish brewers’ association as the government’s formal position (see Gornall, 2014).

Aware of the importance of a favourable reception for the policy among the Commission and other member-states, and the significant efforts being expended by the industry to try to shape the process, officials from the UK and Scottish governments also engaged policy actors at the EU level in attempt to secure support for MUP. As one UK civil servant commented, they succeeded in engaging the health actors within the Commission in order to shape its overall position on MUP:

There were a number of meetings with the Commission, really to encourage them to understand the policy. There was a particular big meeting, I remember, [. . .] with people from different parts of the Commission, not just the health people. And I would say they started off quite negative, and a lot of them were people working with industry. A lot of them it was very noncommittal but emotionally rather negative, but that changed gradually over time. They became much less negative. I think the health people in the Commission did a really good job of really helping for the Commission to be neutral on the issue. And [. . .] by the time the European Court hearing in Luxembourg, the Commission were really pretty neutral, if not slightly positive in some areas, so that was quite a success for UK diplomacy.

Similar views were articulated by counterparts in the Scottish government, who detailed meetings with competition, trade and heath DGs, and underlined how supportive DG Sanco (health) had been on MUP:

We were able to develop relations with individual desk officers and senior managers in DG Sanco to be able to share with them our intelligence and understanding, partly so that they were able – if they felt it useful in their own discussions within the Commission – they were able to use that. But also on occasions they were able to, kind of, ‘raise an eyebrow’ at us that such and such an argument wouldn’t work very well or that it was a good argument and we should go away and make more of it. So, they were able by one means or another to help us through that process, to hone the nature of our arguments.

Claims about the legality of MUP played into domestic political debates and were taken up by opposition MSPs opposing the SNP’s flagship policy (see Holden and Hawkins, 2012). Attempts to shape perceptions of the legality of the policy was one possible way of both influencing public opinion and seeking to undermine political commitment and unity within the government. By highlighting the degree of opposition to the policy at the notification stage, industry actors hoped that the government may be forced to abandon or amend their policy. Ultimately, however, the TSRD process itself did not prove a barrier to the introduction of MUP. The Commission issued a detailed opinion on the case on 26 September 2012, with the Scottish government responding on 21 December 2012 and confirming plans to move forward with MUP. Thus, while the TSRD process created political barriers which the Scottish government had to overcome, and provided an opportunity for various actors to shape the development of the policy, MUP emerged from this process largely intact. However, this was not the only avenue open to industry actors at the EU level through which to seek to prevent the implementation of MUP and, as the policy process developed, they adapted their strategy to respond to the changing circumstances.

## Legal challenges and industry opposition at the CJEU

In July 2012, just weeks after the Scottish government had notified the Commission of its proposed level of MUP under the TSRD notification process, the SWA launched its bid for judicial review of the policy. While the Scottish government had foreseen the possibility they were taken aback by the timing. As one Scottish government respondent commented:

It was perfectly plain that a legal challenge was coming. It came earlier than we had anticipated. We were . . . hard at work with EU processes around the technical standards directive and I think from recollection our best assessment was that the SWA wouldn’t begin litigation, wouldn’t begin judicial review actually, until either that technical standards process had played out fully or at least that it had played out rather more.

Thus, the Scottish government was forced to deal with the considerable challenge of managing not just the domestic political controversy around MUP and the TSRD notification processes at the EU level, but found itself having to respond at the same time to an industry challenge to the legality of the policy. The initiation of legal challenges can also be seen as a form of ‘modal’ venue shifting by which the terrain of the policy debate was moved from the political to the legal domain in which industry actors were more likely to be able to stymie the introduction of MUP.

As part of the CJEU process, member states are able to submit opinions to the Court on the case. This provided a further opportunity for member-states to articulate their positions and concerns about the policy and for the industry to influence the policy process. While, as under the TSRD, member states do not have any formal powers to influence the Court’s judgement, their opinions shape the context in which judgements are made. As one Scottish government civil servant commented, there was a need to enhance political buy-in across the EU:

We didn’t want the optic, the understanding, to develop that we were wholly isolated. It was these odd English-speaking people off the northwest shores of continental Europe that thought this was okay and everybody else realised it was nonsense. So, we spent then quite a number of months [. . .] going round capitals where we thought we could do one of two things, we could get people who were in the first round of responses against us and turn them into silent or neutral or we could get the people who we thought were neutral to come in on our side.

The Scottish government enjoyed a considerable degree of success in changing negative perceptions of the policy which helped to secure the passage of MUP into law. The realisation that the wider political context matters for CJEU decision in health is also in keeping with previous studies of the Court’s ruling on tobacco and alcohol policy issues (Holden and Hawkins, 2018).

As with the TSRD process, industry actors used the various court hearings and judgements to shape perceptions about the legality of the policy, and the degree of support it enjoyed, in order to undermine the policy. Alcohol NGOs sought to counter this strategy through similar public messaging campaigns:

I think the biggest thing was that they just kept on kind of claiming victory throughout [. . .]. I think that was a deliberate campaign to get the politicians to drop it.

While political timetables at the domestic and EU levels are beyond the control of the industry, the decision of the Scottish government to undertake notification with the Commission was influenced by industry pressure and the prospect of further opposition. The decision to instigate a formal legal challenge at the earliest opportunity, when the government was still engaged with the TSRD process is in keeping with previously identified strategies of the tobacco industry to engage in multi-level, multi-dimensional strategies to prevent unfavoured policies (Hawkins et al., 2018). This approach imposes significant challenges for governments with finite resources and diverts time and resources from other policy issues.

## Co-operation between the Scottish and UK governments

Respondents suggested that the Scottish government was perhaps underprepared for the level of engagement needed to support MUP at the EU level during the TSRD and judicial review processes, having already expended significant efforts to secure the passage of the legislation through the Scottish Parliament. For example, one Scottish government actor commented:

My reading of it was that [. . .] because the domestic fight had been so bloody and unpleasant, that was where all the focus had been, and there was little, very little, European preparation and [. . .] not enough understanding of . . . how Brussels ticked and the factors that would make a difference because we had been so domestically focused on the 2010 legislation.

However, they received significant support from the UK government throughout the MUP process. As a Westminster civil servant familiar with the process commented:

Yes, there was [an exchange of knowledge and experience between the Westminster and Edinburgh governments], particularly once the UK government wanted to do minimum unit pricing, then the engagement was very close, and something that’s not really been written about much is again we partnered with the Scottish Government in approaching the EU [. . .] We continued that work even after we shelved the policy for England, so we were still working with the Scottish Government on the legal case to support them.

The referral of the legal challenge to MUP from the Inner House of the Court of Session to the CJEU for a preliminary ruling on the relevant points of EU law had implications not just for the Scottish government, but also for the UK government as the formal representative of the member-state in question. This necessitated close co-operation between policy-makers in London and Edinburgh, even after the UK government had decided not to pursue MUP in England. As a Scottish government civil servant commented:

We had the technical standards directive going on, we were in the Outer House (Scottish appeal court which referred the MUP case to the CJEU). I had these bilateral engagements directly with Number 10 but also with the Health Department and to an extent with the Home Office as well so there were lots of balls in the air that we were all trying to get to work for us.

According to a UK government civil servant, the UK and Scottish governments performed differing but complimentary functions, and worked closely together to discharge them:

At each stage in the domestic courts and the European Courts, both the Scottish Government and the UK Government, we briefed our own teams of lawyers, so we had our own barristers in court, and the Scottish government lawyers were arguing more on the precise merits of the policy [. . .] and the UK government more on the legal principles, not so much is the 50p minimum unit price effective. That really fell squarely behind the Scottish government. So, we partly had to make sure we were in tandem with what each other was saying, but really be on top of the legal issues, so that was really our role.

The fact the Scottish government, as noted above, were fighting the MUP issue on multiple fronts posed a significant call on resources, which colleagues at Westminster attempted to alleviate by managing aspects of the process which overlapped with their areas of competence. Most obviously, this occurred in the context of the legal challenge in which the UK government was a co-litigant with the Scottish government:

Yes, so we were, where there were some issues where we couldn’t prepare for every question, so we were sort of conferring with our lawyers during the hearing on some of the questions. So, actually the hearing was an interesting date in the European Court, it was the day before the general election in 2015. . . yes, that’s right, so it was interesting because during the election campaign Jeremy Hunt had made a statement about the Conservative party would never bring in a minimum unit price, which was potentially a bit undermining of the UK government’s case, but anyway, we just treated that as a party statement not a government statement.

The evidence of co-operation between the Scottish and UK government underlines the importance of context in shaping the political dynamics of policy debates within systems of multi-level governance. While industry actors were identified above as attempting to play off the Scottish and UK levels of decision making, and the varying policy priorities of different ministries, against one another in processes of venue shifting, once the policy processes shifted to the EU level, the Scottish and UK governments worked closely together in navigating the EU policy and legal processes. This demonstrates the importance of cooperation between key policy actors at different levels to secure policy change within systems of multi-level governance.

Despite the co-operation which existed between the Scottish and UK governments in the context of the EU-level policy and legal processes, officials in the Scottish government recognised that governmental action alone would be insufficient to explain the objectives and rationale for MUP to key actors in the EU institutions and national delegations. Consequently, they enlisted the support of alcohol NGOs to advocate for the policy via their affiliate associations in Brussels and equivalent bodies in other member-states.

## NGO advocacy in Brussels

During both the TSRD process and CJEU hearings, it became evident to policy actors in both London and Edinburgh that the objectives of MUP were often misunderstood by EU policy actors outside health. Previous studies of trade and health policy making demonstrate how trade negotiators (understandably) define issues solely in the terms of their own specialism without consideration of their wider social impact, including on health (Hawkins and Holden, 2016). In the case of MUP, respondents identified that national health ministries and health actors were often excluded from member-state consultations and that this tended to be the case in those countries which opposed MUP. Others that had more joined up policy systems in which health actors were involved were more favourable to MUP, and/or more open to persuasion of its merits by policy advocates and the Scottish and UK governments.

Bypassing health actors meant MUP was likely to be seen by member states in narrow economic terms; as a form of trade restriction or protectionism, especially in member states with a domestic alcohol-industry including agricultural interests (e.g. viticulture). As one Scottish health NGO respondent commented:

I have good reasons to believe that policy was at times framed as being a protectionist measure, to protect British industry against competition. So, for instance, we were aware that there was a briefing paper from the industry that said that this was a measure to protect British owned supermarkets against [foreign competitors]. [. . .] And so the argument was that, you know, English wine is expensive and won’t be affected by minimum unit price, and wine from other parts of Europe, some of that came in under MUP. [. . .] It took me a while to understand that, because [. . .] I had approached it purely as a kind of health issue [. . .]. So, communicating and conveying the health reasons behind this, I think, was important in debunking that.

The need for national governments to protect domestic economic interests in the EU context thus offered important opportunities for industry actors to influence national governments and to promote their narrow business interests as being in the national interest. The lobbying strategy in other member states thus mirrors the divide and rule tactics employed within the UK context to try to play off trade and economic ministries against health (Baggott, 1990).

During the course of the MUP process, the Scottish government realised that they needed support in undertaking policy advocacy work at the EU level. It sought the assistance of Scottish health NGOs to build support for MUP via their networks in Brussels and member-state capitals via their trans-national networks. This involved NGOs accompanying Scottish government delegations to EU capitals to explain the policy, leveraging their connections to local alcohol and health NGOs to engage policy makers. In addition, it involved working in Brussels via EU-level civil society organisations (e.g. the European Public Health Alliance (EPHA) and Eurocare) in order to influence both the institutions (i.e. the Commission) and national delegations. As one NGO actor explained:

I think the Scottish Government [. . .] understood that it was important for us to be able to put the message out in Europe about what this policy was about, and to communicate with other member states about that. [. . .]. So [NGOs] spent about a year going back and forward to Brussels, working with Eurocare on a whole range of things [. . .]. But basically, it meant that when the case was actually referred to the European Court, I had a whole load of links in Brussels and with the NGOs who were based there, so it was quite easy to get them to kind of come together to support us on this policy.

The importance of NGO networks was similarly identified by a UK civil servant:

we also met some of the NGOs in Brussels as well to try and get them to support us [. . .] lobbying some of the other member states’ representations in Brussels too, because we did quite a big job along with the Scottish government about thinking about which member states were hostile, which were on the fence, and which were supportive, so that was part of the lobbying effort that went on for quite a long time.

In particular, the affiliation of Scottish and UK NGOs with pan-European bodies, and their ability to draw on their existing policy networks, was vital in gaining access to key policy actors, including the Health Commissioner. Through these channels, Scottish advocates were able to put pressure on Commission officials to support, or at least refrain from opposing, MUP. As one NGO respondent commented:

But the first time we met with [the Health Commissioner], he was saying, oh yeah, your minimum pricing policy, I absolutely love it, it’s great, I really, really support it. And I was saying, well, why are your officials kind of opposing it, why are all your opinions about it being really unhelpful? Oh, you know, I do my best and I’m only a small part of it. I said, you know, that’s not good enough.

Both civil servants and NGO respondents identified this advocacy work as important in shaping how the policy was perceived at the European level, and thus in overcoming potential objections. This in turn was a key dimension in successfully navigating industry legal challenges to secure the introduction of MUP. The case of MUP thus demonstrates the importance of policy networks and the multi-level structure of civil society organisations within the EU in ways which mirror and the political structures which they seek to influence.

## Discussion and conclusion

The dynamics of policy making in the United Kingdom have been greatly affected by EU membership and, since 1999, the devolution of power to sub-state parliaments and governments such as those in Edinburgh. Previous studies on the Europeanization of (Bache and Jordan, 2006), and the post-devolution consequences for, the British state (Cairney, 2011) identify how in many areas UK policy-making now occurs within a complex system of multi-level governance, which varies in composition, effects and outcomes across policy issues and decision-making forums. These include a small number of studies on health policy and the regulation of health harming industries in this context (Asare et al., 2009; Cairney, 2007; Cairney et al., 2011; Katikireddi et al., 2016) and the emergence of a distinctively Scottish ‘policy style’ (Hawkins and Holden, 2012; Keating et al., 2009).

The current article adds to this body of work by offering a case study of the dynamics of policy-level governance in a key area of health policy in which decisions taken at the sub-state, state and supranational levels are highly inter-connected and are often opposed by well-resourced and highly sophisticated policy actors in the form of the alcohol industry. In addition, this study builds on previous studies of multi-level governance through the application, and further refinement, of additional concepts such as venue shifting which serve to further elucidate the dynamics of policy making across governance levels.

The interconnectedness of policy making at different levels of abstraction means decisions taken in one setting have often far-reaching ramifications in others. This creates both opportunities for policy entrepreneurship as occurred with MUP in Scotland, and ‘spillover’ from one jurisdiction to another. MUP followed another earlier key public health innovations such as smoke-free public spaces, in that it was the devolved administration in Scotland which led the way in the development of the policy which led similar measures to come onto the agenda in elsewhere in the United Kingdom, though interestingly in that case Scotland followed developments elsewhere in the EU.

As well as creating additional capacity for policy development, systems of multi-level governance potentially create new veto points at which policies may be challenged and blocked, and can thus make policy change harder to achieve. Sophisticated and well-resourced political actors, such as TNCs, understand this and attempt to use their expertise to prevent unfavoured policies in multiple contexts. In the case of MUP, policy co-ordination mechanisms, such as the TSRD notification procedure, and legal protections under EU law (i.e. the right to judicial review) were identified as potential veto points through which the Scottish government’s MUP proposals had to pass, which industry actors sought to exploit in order to prevent, or at least slow, its implementation.

The findings here are in keeping with previous studies on tobacco and alcohol policy, which identified how systems of multi-level governance can create additional opportunities for TNCs to influence decision-making processes through venue shifting and multi-dimensional lobbying strategies (Hawkins and Holden, 2016; Hawkins et al., 2018; Holden and Hawkins, 2012, 2018). In the case of MUP, this study demonstrates that venue shifting took three forms: (1) intra-institutional venue shifting sought to engage policy actors within the Scottish and UK governments, as well as within the Commission, who were business oriented and may seek to marginalise health actors which were more supportive of MUP; (2) multi-level venue shifting sought to shift the locus of the decision-making from Scotland to the United Kingdom and/or EU levels; and (3) modal venue shifting sought to shift decision making from the political to the legal realm, using judicial review to overturn the adopted policy. Industry actors enjoyed limited success in their efforts to shift policy debates to London or play off UK level policy-makers against those in Scotland in the policy sphere. The enjoyed far greater success shifting decisions to the EU level and into the judicial sphere. Although this was ultimately insufficient to prevent implementation, further opportunities for influence and delay were secured.

The distinction between these types of venue shifting is largely analytical as, in practice, they were closely related in this case. Different aspects of industry strategies were interconnected and overlapped. For example, the alcohol industry used the threat of legal challenges to ensure the Scottish government went through the TSRD procedure, thus creating a further veto point, which the legislation had to navigate. This offered industry actors the possibility to use expertise of EU policy making and alliances with EU level actors and member states to stall the legislation in this new venue.

Modal venue shifting placed significant demands on the Scottish government’s resources. The political and economic costs incurred during the MUP process has had a ‘chilling effect’ on policy debates, with significant inertia in subsequent alcohol policy development in Scotland, as well as being implicated in the ongoing delay in enacting MUP in England (see also Hawkins and McCambridge, 2020b). With such considerations in mind, the industry strategies identified here must be viewed as being at least partially successful.

Alcohol industry actors expended significant resources engaging national and EU level policy actors during both the TSRD and CJEU consultative processes, and pursuing the legal process to the very last possible venue: the UK Supreme Court. This demonstrated a nuanced understanding of the policy context and the multiple points of engagement and opportunities available. It also placed significant pressure on the Scottish government, which was able to draw on the UK government for support, particularly in the context of the legal challenges in which the UK government, as the representative of the EU member state, was a litigant. The levels of support achieved in this instance should not, however, be taken for granted, especially as the policy of the UK government at that time was (and remains) not to proceed with MUP in England.

Given the scale of industry investment, resisting industry influence required co-ordinated activity by policy makers at different levels, and an alliance with civil society actors including health advocates. Well-developed networks and collaborative working relationships between health policy actors in Edinburgh and London (and later Brussels) were able to counter industry efforts to oppose MUP in multiple forums. The Scottish and UK governments worked effectively together, in alliance with NGOs, to counter industry attempts to stymie MUP at the EU level. These findings on alcohol policy align with the findings of previous studies of tobacco advocacy, highlighting the importance of integrated governmental and civil society action across policy settings to counter industry opposition (Hawkins et al., 2018, 2019).

In conclusion, systems of multi-level governance such as the United Kingdom and the EU offer institutional structures which can facilitate the rapid transfer of innovative policy ideas such as MUP both horizontally (e.g. between EU member states) and vertically (i.e. between sub-state, and state-level polities). On the other hand, they extend the range of veto points and the resources required of both state and sub-state actors to implement policy innovations, particularly in the face of concerted opposition by transnational corporations. As such, they create both opportunities and threats for public health actors seeking to implement effective health policies and industry actors seeking to resist them. This article demonstrates how theories of multi-level governance, and the concepts of veto points and venue shifting, provide us with a conceptual vocabulary with which to interrogate the forms of policy influence being exercised by trans-national corporations through detailed case studies of policy debates such as MUP. In addition, it provides important new insights into the nature of alcohol industry political strategy.

## Notes

1. Since 2016 The Department for Business, Energy & Industrial Strategy.
